# Gene Expression Profiling of Gliadin Effects on Intestinal Epithelial Cells Suggests Novel Non-Enzymatic Functions of Pepsin and Trypsin

**DOI:** 10.1371/journal.pone.0066307

**Published:** 2013-06-18

**Authors:** Amarjit Parmar, Dario Greco, Jarkko Venäläinen, Massimiliano Gentile, Emma Dukes, Päivi Saavalainen

**Affiliations:** 1 Research Programs Unit, Immunobiology, and Haartman Institute, Department of Medical Genetics, University of Helsinki, Helsinki, Finland; 2 Department of Bioscience and Nutrition, Karolinska Institutet, Stockholm, Sweden; 3 Department of Pharmacology and Toxicology, University of Eastern Finland, Kuopio, Finland; 4 Genome Informatics Unit, University of Helsinki, Helsinki, Finland; Boston University Goldman School of Dental Medicine, United States of America

## Abstract

Gliadin triggers T-cell mediated immunity in celiac disease, and has cytotoxic effects on enterocytes mediated through obscure mechanisms. In addition, gliadin transport mechanisms, potential cell surface receptors and gliadin-activated downstream signaling pathways are not completely understood. In order to screen for novel downstream gliadin target genes we performed a systematic whole genome expression study on intestinal epithelial cells. Undifferentiated Caco-2 cells were exposed to pepsin- and trypsin- digested gliadin (PT-G), a blank pepsin-trypsin control (PT) and to a synthetic peptide corresponding to gliadin p31-43 peptide for six hours. RNA from four different experiments was used for hybridization on Agilent one color human whole genome DNA microarray chips. The microarray data were analyzed using the Bioconductor package LIMMA. Genes with nominal p<0.01 were considered statistically significant. Compared to the untreated cells 1705, 1755 and 211 probes were affected by PT-G, PT and p31-43 respectively. 46 probes were significantly different between PT and PT-G treated cells. Among the p31-43 peptide affected probes, 10 and 21 probes were affected by PT-G and PT respectively. Only PT-G affected genes could be validated by quantitative real-time polymerase chain reaction. All the genes were, nonetheless, also affected to a comparable level by PT treated negative controls. In conclusion, we could not replicate previously reported direct effects of gliadin peptides on enterocytes. The results rather suggest that certain epitopes derived from pepsin and trypsin may also affect epithelial cell gene transcription. Our study suggests novel non-enzymatic effects of pepsin and trypsin on cells and calls for proper controls in pepsin and trypsin digested gliadin experiments. It is conceivable that gliadin effects on enterocytes are secondary mediated through oxidative stress, NFkB activation and IL-15 up-regulation.

## Introduction

Celiac disease (CD) is an autoimmune disorder caused by the ingestion of gluten present in wheat, barley and rye. It is the alcohol soluble gliadin fraction in gluten which triggers CD in genetically susceptible individuals, i.e. who are HLA-DQ2 or –DQ8 positive [1]–[3]. The screen detected prevalence of CD is approximately 1% in Caucasians and so far the only cure is a strict adherence to gluten-free diet (GFD) [4], [5]. The two hallmarks of CD are villous atrophy and crypt-cell hyperplasia in the small intestine and individuals suffering from CD display a wide range of symptoms, where diarrhoea and malabsorption are only a few to mention [6]–[8].

The proteolytic cleavage of gliadin by digestive enzymes in the stomach and intestine produces an array of distinct but overlapping peptides [9]. Some of these peptides are termed immunodominant, as they can activate the adaptive immune system [9]. Pathogenesis in CD is driven by T-cells which recognize selected immunodominant, deamidated gliadin peptides, such as p57-68, presented by the disease associated HLA-DQ2 or DQ8 molecules on antigen presenting cells [10]–[12]. The T cell activation and mucosal inflammation persists as long as gluten is present in the lamina propria beneath the epithelial layer. Thus, the presence of gluten in the mucosa is a prerequisite for the activation of gluten-reactive T cells and the ensuing inflammation. Another subset of gliadin-derived peptides is termed cytotoxic and these peptides activate the innate immune system which precedes the activation of adaptive immune system in the lamina propria [9], [11], [13], [14].

The gliadin-derived cytotoxic peptides affect the gut in different ways. In experiments on cultured epithelial cells, gliadin peptides have been shown to induce oxidative stress, rearrangement of actin cytoskeleton and impairment of epithelial tight-junction assembly [14]–[17]. Studies by Barone *et al*. showed that gliadin peptides interfere with endocytic vesicle maturation and also promote cell proliferation by prolonging epidermal growth factor receptor (EGFR) activation [18]–[20]. Furthermore, some toxic gliadin peptides (e.g. p31-43 or p31-49 peptides of gliadin) have been reported to induce apoptosis in gut epithelial cells [21]-[23]. These effects are thought to be direct effects of gliadin peptides. Interestingly, the immunodominant peptides (e.g. p57-68) have been shown in these studies to be unable to mimic the effects that the cytotoxic peptides (e.g. p31-43 or p31-49) have on enterocytes. Gliadin peptides must cross the epithelial barrier and enter the mucosa to activate T cells. Several mechanisms (paracellular and transcellular) of gliadin transport across the intestinal epithelial barrier have been suggested [19], [24]–[27]. In addition, some gliadin peptides have been shown to bind to the chemokine receptor CXCR3 on the surface of epithelial cells and induce tight junction permeability [27]. Enterocyte apoptosis caused by gliadin peptides is also considered a potential mechanism of gliadin introduction into the mucosa. The intestinal epithelial barrier is impaired in CD and as luminal antigens are present in close proximity to enterocytes, an interaction between the two is feasible. Despite a multitude of documented effects attributed to cytotoxic gliadin peptides, the molecular mechanisms through which these effects are brought about remain obscure. No study has thus far been undertaken to study the changes in enterocyte gene expression as a result of their interaction with gliadin peptides. A transcription profile of epithelial cells exposed to gliadin may not only help us identify certain receptor-associated signalling pathways but may also help us understand other gliadin mediated effects. Epithelial cells form the first line of defence against environmental antigens. Thus, understanding the exact mechanism by which gliadin peptides affect enterocytes is crucial to our understanding of early stages in CD onset and to the discovery of novel biomarkers for improved diagnostics and therapy targets of CD.

In this study, we sought to answer whether gliadin peptides have any direct effect on intestinal epithelial cell gene transcription, also suggestive of potential cell surface receptor-dependent signalling pathways. To answer these questions we exposed Caco-2 cells to pepsin- and trypsin-digested gliadin (PT-G) and the cytotoxic p31-43 peptide of gliadin for six hours. We used human whole genome microarray chips to obtain a list of differentially expressed genes after gliadin exposure.

## Materials and Methods

### Caco-2 Cell Cultures and Stimulations

Caco-2 cells (American Cell Type Collection, HTB-37, Rockville, MD, USA) were kindly provided by Prof. M. Mäki (University of Tampere, Tampere, Finland). Cells were maintained in minimum essential medium (MEM, 31095-029 GIBCO) supplemented with 1X sodium pyruvate (NaPy 100X, 11360-039 GIBCO), 1% Pen-Strep (Pen-Strep, 15070-063 GIBCO), 1X non-essential amino acids (NEAA 100X, 11140-035 GIBCO) and 10% fetal calf serum (FCS, C-37350 PROMOCELL) in 75 cm^2^ flasks (353136 BD Biosciences) and were routinely split every 5 days. For stimulations, 3×10^5^ cells were seeded in 25 cm^2^ flasks (156367, nunc) in 3 ml MEM+1% FCS and incubated at 37°C with 5% CO_2_ in the air. On the third day of seeding, the undifferentiated Caco-2 cells were washed once with 1X phosphate-buffered saline (PBS). Bovine serum albumin (BSA) was obtained from Sigma Aldrich (Albumin, bovine fraction V powder, A8806-1G Sigma Aldrich) and contained endotoxin at ≤0.1 ng/mg. Lactalbumin was also obtained from Sigma Aldrich (Alpha-lactalbumin from bovine milk, 61289-50MG Sigma Aldrich), however information on endotoxin level in lactalbumin was not available. Pepsin- and trypsin- digested BSA (PT-BSA), pepsin- and trypsin- digested lactalbumin (PT-L), a blank pepsin- and trypsin- control (PT), where no substrate was used for digestion, and PT-G were prepared as described earlier [28]. The pepsin and trypsin enzymes were inactivated by heating after substrate digestion and the inactivation was verified with enzyme activity assay using fluorogenic trypsin substrate Z-Arg-AMC (data not shown). An endotoxin test was performed on PT-G and PT-BSA using a Limulus Amebocyte Lysate (LAL), Pyrogent plus single test kit (cat no. N289-06, CAMBREX). No endotoxin was found in PT-G, but PT-BSA tested positive for endotoxin. Lyophilised PT, PT-BSA, PT-L and PT-G were dissolved in MEM+1% FCS and filter sterilised by passing through 0.22 µm pore filter (SLGS033SS, Millipore). Similarly the synthetic peptides p31-43 (LGQQQPFPPQQPY) and the immunogenic peptide p57-68 (QLQPFPQPQLPY) (New England Peptide LLC, USA) were dissolved in MEM+1% FCS to achieve a final concentration of 150 µg/ml. PT, PT-BSA and PT-L were used as negative controls for PT-G treatment. The immunogenic p57-68 peptide served as a negative control for the p31-43 treatment as it has previously been shown not to have any effect on epithelial cells [11], [19], [20], [29]. The cells were stimulated for six hours with a particular stimulant dissolved in 2 ml MEM+1% FCS. The control cells (MED-CTL) were kept in MEM+1% FCS for six hours. In total, four identical but independent experiments were performed with cells differing in 1–2 passages between each experiment.

### Cell Collection and RNA Extraction

The cells were collected by adding 1 ml 1X trypsin-EDTA (Trypsin 10X, 15400-054 Invitrogen) to the flasks followed by 4–5 min incubation at 37°C. Trypsin was inhibited by adding 1 ml MEM+10% FCS and the cells were collected by centrifugation at 9.600×g for 5 min. The pellet was washed with 500 µl 1X PBS and RNA was extracted using RNeasy Plus Mini kit (74134, QIAGEN).

### Microarray Data Production and Analysis

**Figure 1:**
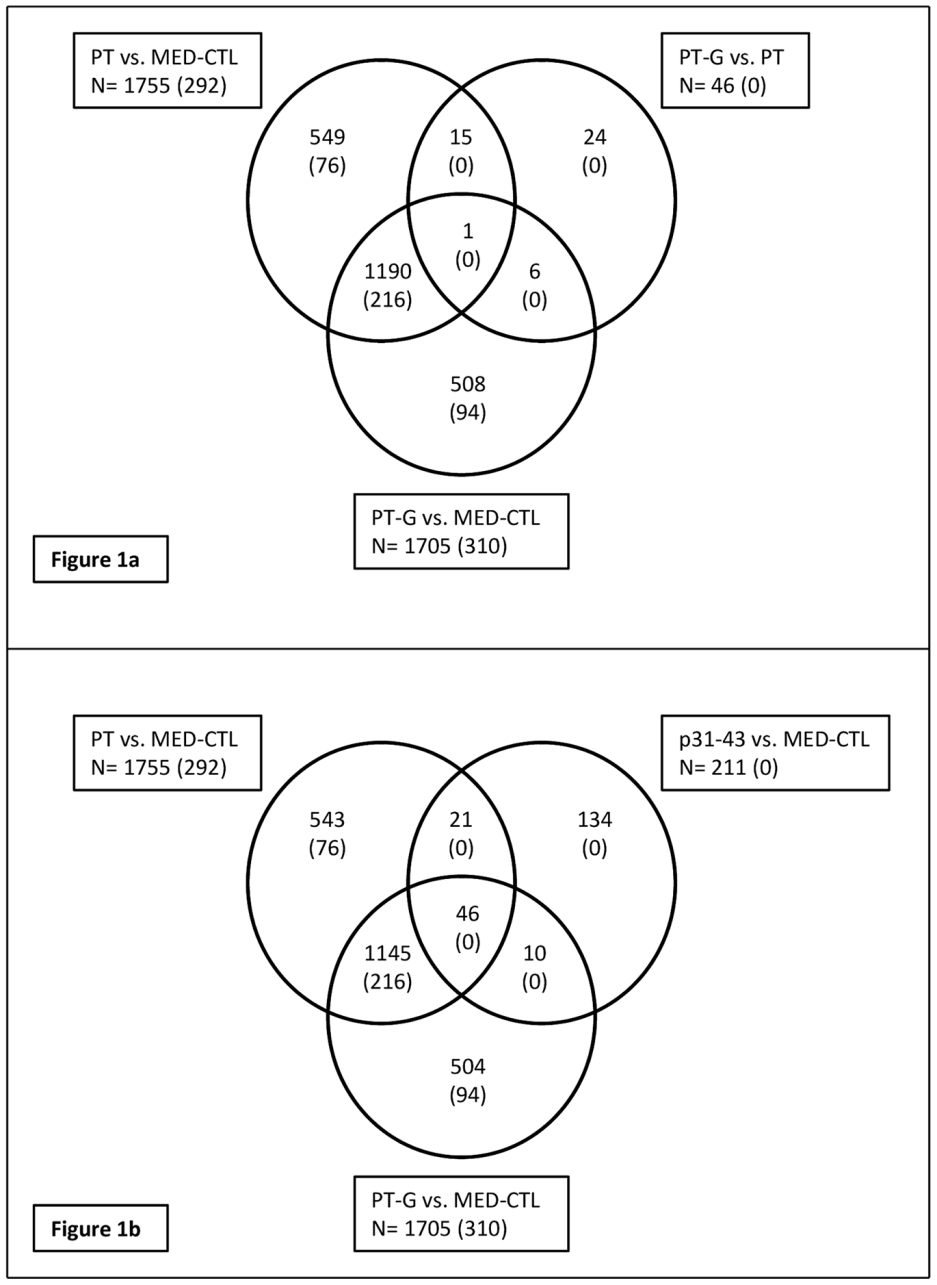
Venn diagrams showing the number of probes differentially expressed in response to pepsin- and trypsin-digested gliadin (PT-G) (Figure 1a) compared to medium control (MED-CTL) and the blank pepsin- and trypsin (PT) control. The probes that were affected by PT treatment compared to MED-CTL are also displayed. Figure 1b shows the probes affected by PT, PT-G and p31-43 peptide compared to MED-CTL. The numbers in parenthesis represent the number of probes obtained after multiple testing correction as described in materials and methods.

RNA from PT-treated, PT-G-treated, MED-CTL and p31-43 stimulated cells was used for hybridisation on Agilent one colour human whole genome DNA microarray chips (4×44K) (Agilent Technologies, Santa Clara, CA). RNA quality for these samples was assessed by bioanalyzer (2100 Bioanalyzer, Agilent) and 600 ng of RNA was used for cDNA synthesis. The cDNA synthesis and array hybridisations were carried out at Biomedicum Genomics Support, Helsinki, Finland. Microarray raw data (.gpr files) were imported into R v. 2.15 [30] and analyzed with the BioConductor [31] package limma [32]. Briefly, after quality check, the microarray probes were filtered and re-annotated according to Gertz *et al*. [33] and their median foreground intensity was normalized with the quantiles method without applying any background correction, as previously suggested [34]. Finally, the probes were tested for differential expression using a linear model followed by moderated t-test [32] for the comparisons of interest. Genes with nominal p<0.01 were considered to be differentially expressed and further considered in the analysis. Correction for multiple testing using Benjamini and Hochberg method [35] resulted in only few statistically significant genes (Figure 1) and therefore the study was mainly based on the uncorrected gene lists. The microarray data has been submitted to Gene Expression Omnibus (accession number: GSE45357).

### Quantitative Real-time Polymerase Chain Reaction (qRT-PCR)

Microarray results were validated by qRT-PCR and five genes which were not affected by PT treatment were selected (Table 1). The genes with high expression level (≥ log2 9.0) and with a fold-change (FC) ≥1.4 in either direction (up-regulation or down-regulation) were prioritized (tables S1, S2, S3, S4). However, *RELA* gene (FC = 1.31) was an obvious exception to this rule and was included for validation by qRT-PCR because of its involvement in the nuclear factor kappa B (NFkB) pathway. The NFkB pathway has been shown to be activated in small-intestinal mucosa of CD patients. [36] Primers for the selected genes were designed using the primer designing software Primer express (Applied Biosystems) (Table 1). 300 ng of RNA was used to synthesise cDNA using TaqMan Reverse Transcription Reagents (N808-0234, Applied Biosystems) as recommended by the vendor. Microarray results for selected genes were verified using Power SYBR Green PCR Master Mix (4367659, Applied Biosystems, 1X SybrGreen PCR mix, 0.6 µM forward and reverse primer each) on 7500 Fast Real-Time PCR System (Applied Biosystems). The gene expression levels were normalised against *beta-2 microglobulin (B2M)* gene expression.

**Table 1:** Primers used for qRT-PCR.

Gene	Forward (5′-3′)	Reverse (5′-3′)
*CEBPa*	GACCCTCAGCCTTGTTTGTACTG	CTGATCGTGCTTCGTGTTCCT
*KLF4*	ACCAGGCACTACCGTAAACACA	GCTCGGTCGCATTTTTGG
*SPRED1*	TGAATGCTGCTACAACAGATGATC	GAAAGTTAACAGTCTATTCTAGGAAACCAA
*MNF1*	CACAGACACCTTGGAAGAGCTTAA	GGGCAAACTTCTCCTGCAGTT
*RELA*	CAGGCGAGAGGAGCACAGA	TGTGTAGCCATTGATCTTGATGGT
*B2M*	GTGCTCGCGCTACTCTCTC	GTCAACTTCAATGTCGGAT

CEBPa: CCAAT/enhancer binding protein alpha, KLF4: Kruppel-like factor 4, SPRED1: Sprouty-related, EVH1 domain containing 1, MNF1: Mitochondrial nucleoid factor 1, REL-A: v-rel reticuloendotheliosis viral oncogene homolog A, B2M: Beta-2 microglobulin.

## Results

### Differentially Expressed Probes in Caco-2 Cells after Stimulation with PT-G and the p31-43 Peptide

Many of the differentially expressed genes are represented by multiple probes on the Agilent microarrays utilized in this study, hence the term probes, instead of genes, is used. The expression of 1705 probes was affected by PT-G treatment compared to the untreated cells (MED-CTL) that were kept in medium for the duration of the stimulation (Figure 1a). However, 1755 probes were also affected by PT-only treatment. In a comparison between PT-G treated cells and PT treated cells, 46 probes were found to be differentially expressed. In cells which were exposed to the cytotoxic p31-43 peptide, 211 probes were differentially expressed compared to the untreated cells (Figure 1b). Of these, 21 probes were also affected by the blank PT treatment and ten probes were affected by PT-G treatment and 46 probes were affected by PT, PT-G and p31-43 treatments compared to MED-CTL. All the probes, along with their expression data, affected by PT, PT-G or p31-43 are listed in tables S1, S2, S3, S4.

### Validation of Differentially Expressed Genes by qRT-PCR

Five genes were selected for validation using criteria described in materials and methods. Fold change (FC) values greater than one indicate up-regulation and those less than one indicate down-regulation. *Kruppel-like factor 4* (*KLF4*) (FC PT-G vs. MED-CTL: 2.0), *sprouty-related, EVH1 domain containing 1* (*SPRED1*) (FC PT-G vs. MED-CTL: 1.66) and *CCAAT/enhancer binding protein alpha* (*CEBPa*) (FC PT-G vs. MED-CTL: 0.71) were affected by PT-G treatment, whereas *mitochondrial nucleoid factor 1* (*MNF1*) (FC p31-43 vs. MED-CTL: 1.91) and *v-rel reticuloendotheliosis viral oncogene homolog A* (*RELA*) (FC p31-43 vs. MED-CTL: 1.31) were affected by treatment with the gliadin p31-43 peptide. The qRT-PCR results are shown in Table 2. The average expression of the PT-G affected genes in the qRT-PCR assay was similar to that of microarrays, thus validating the results. However, similar expression values were also obtained for PT, PT-BSA and PT-L treated cells, which were used as negative controls for PT-G treatment. The microarray results of the p31-43 treatment could not be validated by qRT-PCR. The average expression of *MNF1* and *REL-A* in p31-43 cells was comparable to that of untreated (MED-CTL) and p57-68 peptide treated cells.

**Table 2:** Validation of microarray results for selected genes by qRT-PCR.

qRT-PCR								Microarray	
Gene	MED-CTL	PT	PT-BSA	PT-L	PT-G	p57-68	p31-43	PT-G	p31-43
*CEBPa*	1.01	0.80	0.73	0.66	0.66			0.71	
*KLF4*	1.06	1.71	1.91	1.72	1.78			2.0	
*SPRED1*	1.04	1.48	1.38	1.16	1.50			1.66	
*MNF1*	1.00					0.93	0.84		1.91
*RELA*	1.05					0.99	1.05		1.31

The qRT-PCR results for CCAAT/enhancer binding protein alpha (CEBPa), Kruppel-like factor 4 (KLF4), SPRED1: Sprouty-related, EVH1 domain containing 1 (SPRED1) are based on eight independent experiments. The qRT-PCR results for Mitochondrial nucleoid factor 1 (MNF1), REL-A: v-rel reticuloendotheliosis viral oncogene homolog A (REL-A), are based on five independent experiments. The expression of genes was normalised against Beta-2 microglobulin (B2M) gene The values expressed for different treatments are the average expression values.

### Comparison of Microarray Results with Previous Studies

Several differentially expressed genes in our study were also reported in previously published expression studies performed either on biopsy specimens or enterocytes isolated from biopsies [37]-[39]. These genes together with their expression values are tabulated in Table 3. 13 out of 19 genes were affected by PT treatment, five genes (*CD59 antigen*, *CD59*; *Ephrin B2*, *EFNB2*; *Myosin VI*, *MYO6*; *ETS-domain protein*, *ELK4*; *Prion protein*, *PRNP*) were affected by PT-G only, whereas only one gene (*RNA-binding protein*, *RALY*) was affected by p31-43 treatment. *RALY*, *EFNB2*, *MYO6* and *ELK4* were up-regulated to a similar degree in our microarray experiment and another previously reported study, whereas *CD59* and *PRNP* were up-regulated in our study but were shown to be down-regulated previously.

**Table 3:** Comparison of microarray data with previous studies.

Microarrays				Previous studies			
	PT-G	PT	p31-43	Juuti-Uusitalo *et al.* 2004	Diosdado *et al.* 2008	Bracken *et al.* 2008	Condition
*DLX4*	1.60	1.50		1.95			UCD vs. HC
*DLX4*	1.60	1.50		1.42			UCD vs. TCD
*DLX4*	1.60	1.50		1.37			TCD vs. HC
*JUNB*	3.40	2.90		0.75			UCD vs. HC
*NAB2*	2.00	1.90		0.54			UCD vs. HC
*PROCR*		1.40		1.43			TCD vs. HC
*SLC25A6*		1.80	1.70	1.40			UCD vs. HC
*SLC25A6*		1.80	1.70	1.45			UCD vs. TCD
*RALY*			1.60	1.43			TCD vs. HC
*CD59*	1.40			0.66			UCD vs. HC
*EFNB2*	1.40			1.41			UCD vs. TCD
*MYO6*	1.27			1.27			TCD vs. HC
*HSPA6*	2.60	2.90			1.32		MIII-G+/G-a
*MSI2*	0.75	0.72			0.78		MIII-G+/G-a
*POLD3*	0.66	0.61			0.63		MIII-G+/G-a
*RGS16*	2.70	2.57			0.81		MIII-G+/G-a
*ELK4*	1.37					1.45	Ent. CD vs. Ent. HC
*UBE3A*		0.82				0.77	Ent. CD vs. Ent. HC
*NUDT2*	0.72	0.74				2.21	Ent. CD vs. Ent. HC
*PRNP*	1.31					0.54	Ent. CD vs. Ent. HC

Expression values >1 indicate up-regulation and <1 indicate down-regulation. Condition refer to the experimental condition used in the original study. UCD: Biopsy from untreated CD, TCD: Biopsy from treated CD, HC: Biopsy from healthy control, MIII-G+: Biopsy from Marsh-III stage CD patient on gluten containing diet, MIII-G-: Biopsy from Marsh-III stage CD patient on gluten-free diet, Ent. CD: Epithelial cells isolated form duodenal biopsies of CD patients, Ent. HC: Epithelial cells isolated from duodenal biopsies of healthy controls.

a In the original study, the expression values were given for MIII-G−/G+ and comparable values for MIII-G+/G- have been obtained (1/MIII-G−/G+).

## Discussion

In the current study, the microarray data suggested multiple effects of PT-G on Caco-2 cell gene transcription. However, an effect similar in size was also evident by PT treatment. Our data also suggest that genes which seemed unaffected by PT in the microarray are in fact affected by negative controls, PT-BSA, PT-L and PT, in qRT-PCR. This study was unable to identify genes in epithelial cells which are affected by gliadin. Potential explanations for this outcome may be that the effects exerted by gliadin on epithelial cells are secondary effects, the presence of other cells of the immune system is required for gliadin induced activation of enterocytes, the gliadin effects are so small that very sensitive methods are required to detect them or six hours stimulation time was not optimal.

Nevertheless, multiple effects of gliadin on enterocytes have been shown. Previously, Giovannini *et al*., showed that gliadin peptides induce enterocyte apoptosis via Fas-Fas ligand (FasL) pathway [21], [40]. The authors reported that after PT-G exposure, the cells increased their mRNA expression of *Fas* and *FasL* by 450% and 170% respectively compared to the controls. This increase in Fas/FasL transcription was observed at 6 h, 18 h and 24 h after PT-G exposure. However, neither *Fas* nor *FasL* transcription was affected in our experiment after 6 h, but in cells treated with PT, the expression of a Fas-activated serine/threonine kinase (*FASTK*) was marginally repressed. FASTK is rapidly activated during Fas-mediated apoptosis [41]. Furthermore, *FAST kinase domains 2* (*FASTKD2*) gene, which is believed to be a pro-apoptotic gene [42], was down-regulated by both PT and PT-G treatments. PT treatment also repressed the expression of *caspase 8* (*CASP8*) whose product has been implicated in PT-G mediated apoptosis in enterocytes [21]. This may suggest that PT/PT-G inhibits apoptosis in Caco-2 cells. Nonetheless, several other apoptosis-related genes were affected by both PT and PT-G treatments (tables S1, S3, S4). Thus the effect of PT/PT-G on Caco-2 cells with regard to apoptosis is not clear. Difference in our results and those of Giovannini *et al.* may depend on different cell culturing conditions.

On the contrary, studies on biopsy specimens from treated/untreated CD patients demonstrate that exposure to interleukin (IL)-15 induces enterocyte expression of transferrin receptor (TFR), proliferation marker Ki67 and FAS in treated CD and also apoptosis in untreated CD biopsy specimens [43]. Furthermore, lamina propria mononuclear cells (LMNPs) in biopsy specimens of treated CD patients become IL-15 positive after PT-G treatment and also induced enterocyte apoptosis in a Fas-FasL dependent manner [23], [43]. Similar results have been reported for the cytotoxic p31-43 peptide. PT-G and p31-43 peptide were reported to induce proliferation in epithelial cells which is dependent on both IL-15 and epidermal growth factor receptor (EGFR) activity [18], [20]. Furthermore, p31-43 induces IL-15 dependent enterocyte apoptosis in biopsy samples obtained from untreated CD patients [11]. IL-15 has also been shown to induce enterocyte MICA expression in CD patients [44] and in triggering anti-apoptotic pathway in human intraepithelial lymphocytes (IELs) which can kill IECs [12], [45]. These studies suggest a central for IL-15 in CD pathogenesis. Recently, it was reported that in Caco-2 cells, p31-43-mediated increase in *IL-15* mRNA was detectable only after over-night (ON) treatment [20]. In agreement with this observation, we did not see an enhanced *IL-15* transcription after PT, PT-G or p31-43 treatment for six hours. Gliadin induces oxidative stress in epithelial cells and oxidative stress is known to activate NFkB transcription factor [14], [46]–[48]. Furthermore, it has been shown that NFkB activation is required for *IL-15* induction in rhinovirus infected macrophages [49], [50]. Increased NFkB activity is present in small-intestinal mucosa of CD patients [36]. It is conceivable that enhanced *IL-15* transcription after ON treatment with gliadin peptides is a secondary effect which operates through gliadin induced oxidative stress leading to NFkB activation and subsequent *IL-15* transcription.

Binding to the chemokine receptor CXCR3 by some gliadin peptides was suggested to induce an increase in intestinal permeability [27]. Recently, Caputo *et al*. reported that gliadin peptides p31-43 and p57-68 induce intracellular calcium ion mobilization leading to endoplasmic reticulum (ER)-stress [51]. Whether these effects were mediated by binding of gliadin peptides to a cell surface receptor or by some other mechanism was not addressed. Other non-receptor mediated effects of gliadin on tight junction (TJ) protein disassembly have also been reported [15], [16], [52]. It is possible that gliadin transported through leaky TJs does not affect epithelial cells directly but activate cells of the immune system in the underlying mucosa.

As shown in Table 3, several differentially expressed genes in our study have previously been reported by others [37]–[39]. Our study is not directly comparable to these studies as these studies were performed either on CD biopsy specimens or on enterocytes obtained from biopsy specimens. Interestingly, the biopsy specimens or enterocytes used in these studies were not exposed to PT or PT-G *in vitro*. Some of the genes reported in these studies are affected by PT and PT-G to a comparable degree in our study. This observation suggests that endogenous pepsin and trypsin may affect enterocytes under physiological conditions and warrants further investigation. Nonetheless, these studies suggest that small changes (less than two-fold) in gene expression are likely. Despite the fact that some genes have comparable expression value in our study and these studies, these genes must be independently validated.

Pepsin and trypsin are key digestive enzymes acting in stomach (pH 1.5–2.0) and duodenum (pH 7.5–8.5), respectively. In addition to their role in degrading food proteins into peptides, some other non-digestive functions have also been reported. In a microarray study on laryngeal and pharyngeal epithelial cells, non-acidic pepsin (pH = 7.0) affected the expression of cancer-related genes and also promoted their proliferation [53]. Trypsin is an endogenous activator of protease-activated receptor-2 (PAR2). In another microarray study, the human embryonic kidney cells (HEK293) were exposed to trypsin [54]. Several genes involved in cell cycle regulation, metabolism and mitogen-activated protein kinase (MAPK) pathway were reported to be affected. Of these, two pepsin affected genes and 16 trypsin affected genes were also differentially expressed in our study (data not shown). For all, but two trypsin-affected genes, these changes were in the same direction and of comparable magnitude. This observation suggests that pepsin and trypsin may be enzymatically active in our PT-preparations. This is, however, unlikely as pepsin and trypsin were inactivated by heating after substrate digestion. The inactivation of these proteases was verified with enzyme activity assay using fluorogenic trypsin substrate Z-Arg-AMC (data not shown). Nevertheless, it is possible that these are non-enzymatic effects of pepsin and trypsin and are caused by some epitopes that are generated or left intact during deactivation process. Thus, assays to determine the activation potential of such epitopes prior to their use in experiments may be necessary.

PT is the common denominator between PT and PT-G and a majority of the affected genes were shared between the two treatments, but not by p31-43 treatment. The changes observed in gene transcription cannot be attributed to endotoxin as PT-G tested negative for endotoxin. PT is unlikely to be endotoxin-positive as it did not contain any substrate and was prepared at the same time as PT-G, PT-BSA and PT-L. Another possible reason is that, perhaps, PT-G does not affect enterocyte gene transcription. The changes in transcription we observed could be non-specific generic effects, as they were produced by PT, PT-G, PT-BSA and PT-L alike. However, this study does not provide conclusive evidence for that and other studies with a similar design are highly recommended. Furthermore, mechanisms by which gliadin is transported across the epithelial layer may also have an impact on their effect on enterocyte gene transcription. If gliadin peptides are trapped in endocytic vesicles or complexed with other molecules, they may be unable to affect gene expression [25], [26]. However, cells may respond to oxidative stress caused by the accumulation of gliadin peptides in lysosomes [14].

In conclusion, this study does not lend support to previous studies (performed under similar conditions) which reported direct effects of gliadin peptides on epithelial cells. We cannot conclude from our data that gliadin peptides do not effect Caco-2 gene transcription since it is practically impossible to replicate and verify by qRT-PCR all genes suggested by the microarray. There may be other genes with lower fold-changes that are true positive genes. The provided lists of PT-G and PT-affected gene list may be helpful in selecting genes for further validation. This study highlights potentially novel non-enzymatic roles of pepsin and trypsin and warrants further studies. Nonetheless, this study outlines the need for proper controls for pepsin and trypsin in experiments with digested gliadin or other substrates of these enzymes, similar to the PT-only control used in this study. A PT-only control will be helpful in identifying false positive results and in drawing wrong conclusions in any future studies with digested gliadin.

## Supporting Information

Table S1 **PTG vs. MED-CTL differentially expressed probes.**
(TXT)

Table S2 **p31-43 vs. MED-CTL differentially expressed probes.**
(TXT)

Table S3 **PTG vs. PT differentially expressed probes.**
(TXT)

Table S4 **PT vs. MED-CTL differentially expressed probes.**
(TXT)
